# Large-scale audio dataset for emergency vehicle sirens and road noises

**DOI:** 10.1038/s41597-022-01727-2

**Published:** 2022-10-04

**Authors:** Muhammad Asif, Muhammad Usaid, Munaf Rashid, Tabarka Rajab, Samreen Hussain, Sarwar Wasi

**Affiliations:** 1 Data Acquisition, Processing, and Predictive Analytics, NCBC, Ziauddin University, Karachi, Pakistan; 2 Aror University of Art, Architecture, Design and Heritage, Sukker, Pakistan

**Keywords:** Databases, Engineering

## Abstract

Traffic congestion, accidents, and pollution are becoming a challenge for researchers. It is essential to develop new ideas to solve these problems, either by improving the infrastructure or applying the latest technology to use the existing infrastructure better. This research paper presents a high-resolution dataset that will help the research community to apply AI techniques to classify any emergency vehicle from traffic and road noises. Demand for such datasets is high as they can control traffic flow and reduce traffic congestion. It also improves emergency response time, especially for fire and health events. This work collects audio data using different methods, and pre-processed them  to develop a high-quality and clean dataset. The dataset is divided into two labelled classes one for emergency vehicle sirens and one for traffic noises. The developed dataset offers high quality and range of real-world traffic sounds and emergency vehicle sirens. The technical validity of the dataset is also established.

## Abstract (summary)

**Table:** 

Measurement(s)	Ambulance Siren and Road Noises. (Recordings)
Technology Type(s)	Microphone
Sample Characteristic - Environment	Roads
Sample Characteristic - Location	Pakistan

## Background & Summary

Artificial intelligence (AI) is now extensively used in many classification problems including audio classification. Datasets are the key to any AI algorithm for training and decision-making. Similarly, any audio event-triggered AI algorithm requires a large-scale audio dataset for acoustic detection. AI techniques such as machine learning (ML) and deep learning (DL) for audio event detection and identification are in high demand these days. In addition, the researchers have implemented countless signal processing and AI techniques on the datasets to achieve their research objectives. However, collecting a dataset is a gigantic task, requiring efforts on a larger-scale, time, and resources.

With the availability of large datasets, researchers are making great strides in identifying and understanding audio^1–6^. They have used various audio datasets that are publicly available, providing over a thousand audio clips labelled in multiple categories for different essential sounds, such as clapping, laughter, music, environmental noise, etc. Another large-scale dataset was published^7,8^ that included more than 40 classes of daily life sounds. However, there are different domains of life where data sets are scarce, or there is no precise data set available for study. It creates a massive gap between the dataset’s applications and the researchers. A large  dataset is required to train the data-hungry AI algorithms, while the amount of human effort and resources to develop such datasets is enormous, e.g., as stated in^9–11^, there is no clear, detailed, and labelled dataset available for the ambulance or emergency vehicle siren and the road noises.

Due to the increase in traffic volume, and traffic congestion, roadaccidents have become the norm in the metropolitan cities^12^. It increases the demand for datasets to help control traffic flow and improve emergency response time, especially for fire and health-related incidents. This research effort aims to develop a specific dataset for the sound of emergency vehicle sirens. This research work describes the development of an audio  dataset, a voice that offers a wide range of real-world traffic sounds and emergency vehicle sirens.

The “ambulance siren” class is not richly interpreted in other papers. For instance, after extensively reviewing the literature^13–18^, the google AudioSet^13^; the complete dataset is in video format and for downloading, you have to download videos, then convert all of them into audio. Even the data is not in the uniform length (AUDIO). This is such an extensive process and require large efforts. Similarly, the Urban Sound Dataset^14^; to access the dataset first, you have to fill the request form and wait for the permission. Additionally, all the siren files are of only four second duration only. While another dataset^15^ contains only two kinds of sounds: sirens and horns while discarding the other road noises. The audio files are of very small lengths 0.5 s to 2.5 s only and with no any specific and concrete information about the dataset and how many files have been collected.

Referring to the dataset^16^ which is categorized into two classes: Normal and Anomalous audios while anomalous class is sub divided into siren, horns and pedestrian audios and having duration of one second only. To remedy this short coming another dataset which is also published by the UrbanSound^17^ which is versatile and having total of 10 classes, including the siren class. However, the audio duration is of 0 to 4 seconds only. Another dataset^18^ published and which is publicly available, contains two classes namely: Positive and Negative class while positive class contain all the audio files of sirens, and fire vehicles. The dataset is having only 47 audio samples of sirens of 2 second duration only which is relatively considered as smaller dataset.

**Fig. 1:**
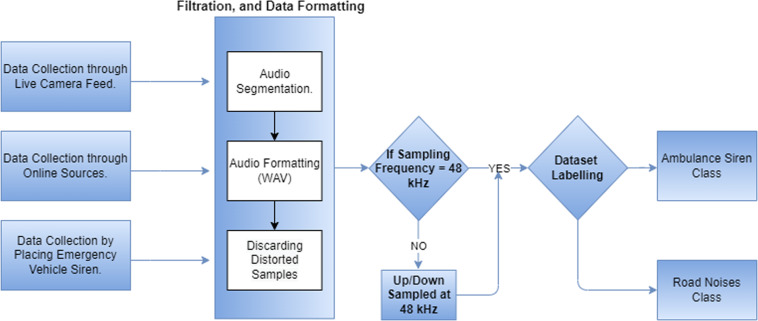
The framework used for the dataset development; This figure explains and summarizes the dataset collection process and techniques.

In this work, a dataset has been developed using two labelled classes: one for emergency vehicle sirens and the other for the traffic noise without the sound of emergency vehicle sirens. The labelled dataset enables us to define the data required for training and testing. The developed dataset is available to the scientific community and is ready for training. The scientific community can use this dataset to create an intelligent audio event detection system in a real-world environment. The complete database development process is shown below in Fig. 1.

## Methods

This section discusses the methodology used to collect sound data and extract its features. This research is performed at National Centre for Big data and Cloud Computing, Ziauddin University (NCBC-ZU), Karachi, Pakistan. Sound data is collected using various techniques to build a dataset: (1) Data collection through mic integrated HD Camera, (2) Online Sources, and (3) Placing Emergency Siren manually.

### Data collection procedures

**Fig. 2:**
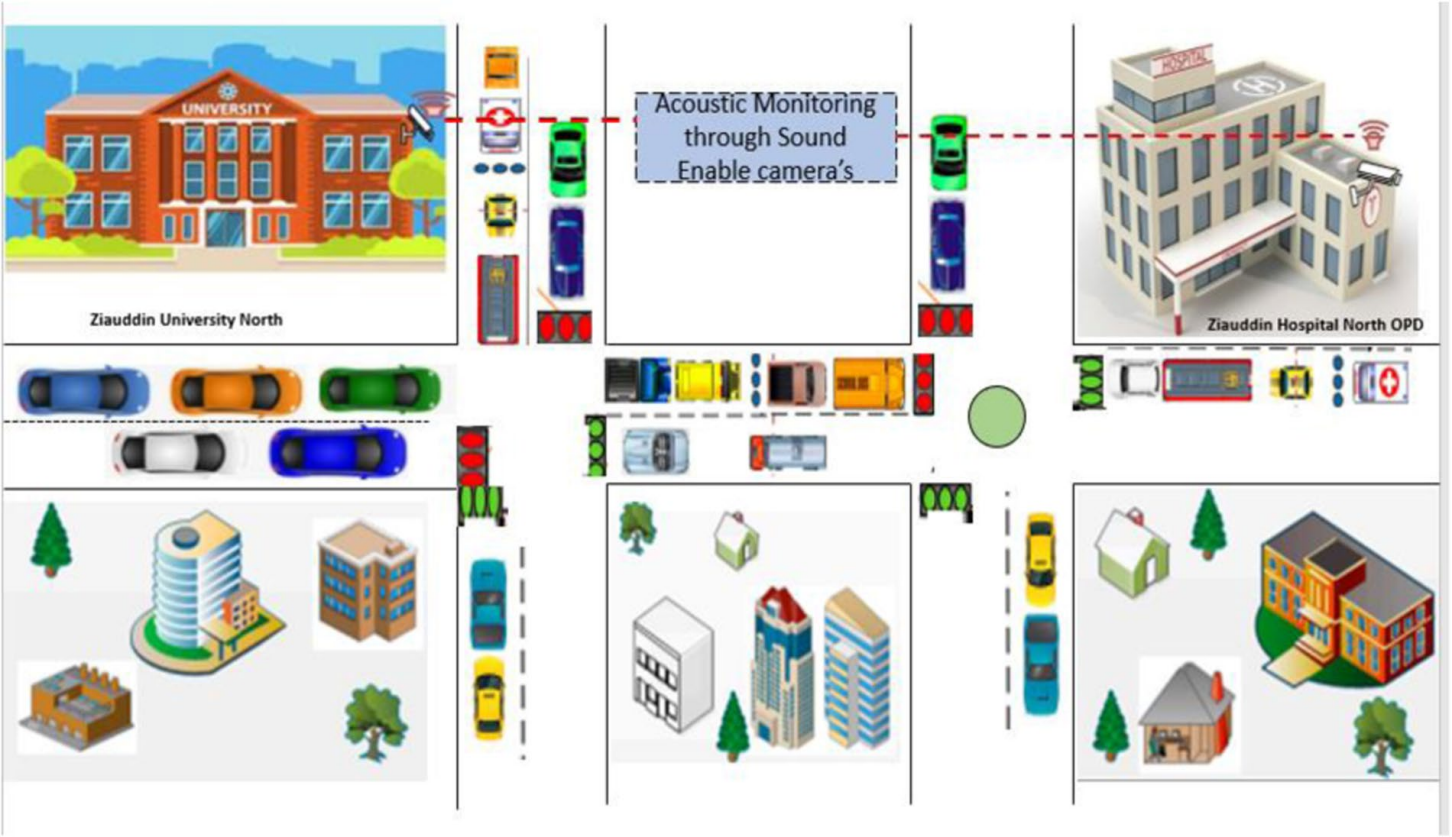
The Architecture of data recording process through Live Camera Feed; This figure shows the pictorial representation of different locations from which live feed has been taken and converted into audio files for database development.

Various methods have been implemented to build a database. Firstly, a microphone mounted on the HD camera is used to capture the sound of an ambulance on the roads. NCBC-ZU lab has installed microphone-integrated HD cameras at twenty different locations in Karachi. However, only four locations have been selected for data collection. The first location is a road in front of Ziauddin University, Faculty of Engineering, Science, Technology and Management (ZFESTM), Block B, North Nazimabad, Karachi (Latitude 24.927920124766707, Longitude 67.04174087630918). The location is selected because it is located between two hospitals within one kilometre. The second location is a road in front of Dr Ziauddin Hospital, North Nazimabad, Karachi, Pakistan (Latitude 24.924592631257386, Longitude 67.04675122325636). The third location is a road in front of Dr Ziauddin Hospital, Clifton, Karachi, Pakistan (Latitude 24.817493704317904, Longitude 67.00745787684244). The reason is obvious because both roads are in front of tertiary care Hospitals. The fourth location is the SSUET site, an entrance road of Sir Syed University of Engineering and Technology, Gulshan-e-Iqbal, Karachi (Latitude 24.91577878677469, Longitude 67.09204418684436), the main centre of the city. Figure 2 represents the architecture of the data collection process through voice-enabled cameras.

The 24 hours video was checked for occurrences of an ambulance siren, the part which contained the siren was trimmed. The clipped videos were then converted into audio files using video to mp3 converter software. The emergency siren audio files were labelled in the emergency vehicle siren class. In contrast, all other sounds and noises on the road were labelled in a separate class. In addition, ambulance sirens were also collected through the same process from other IP-based cameras. Hence, the dataset contains two types of classes: emergency siren sounds and all other sounds found on real-world roads.

**Fig. 3:**
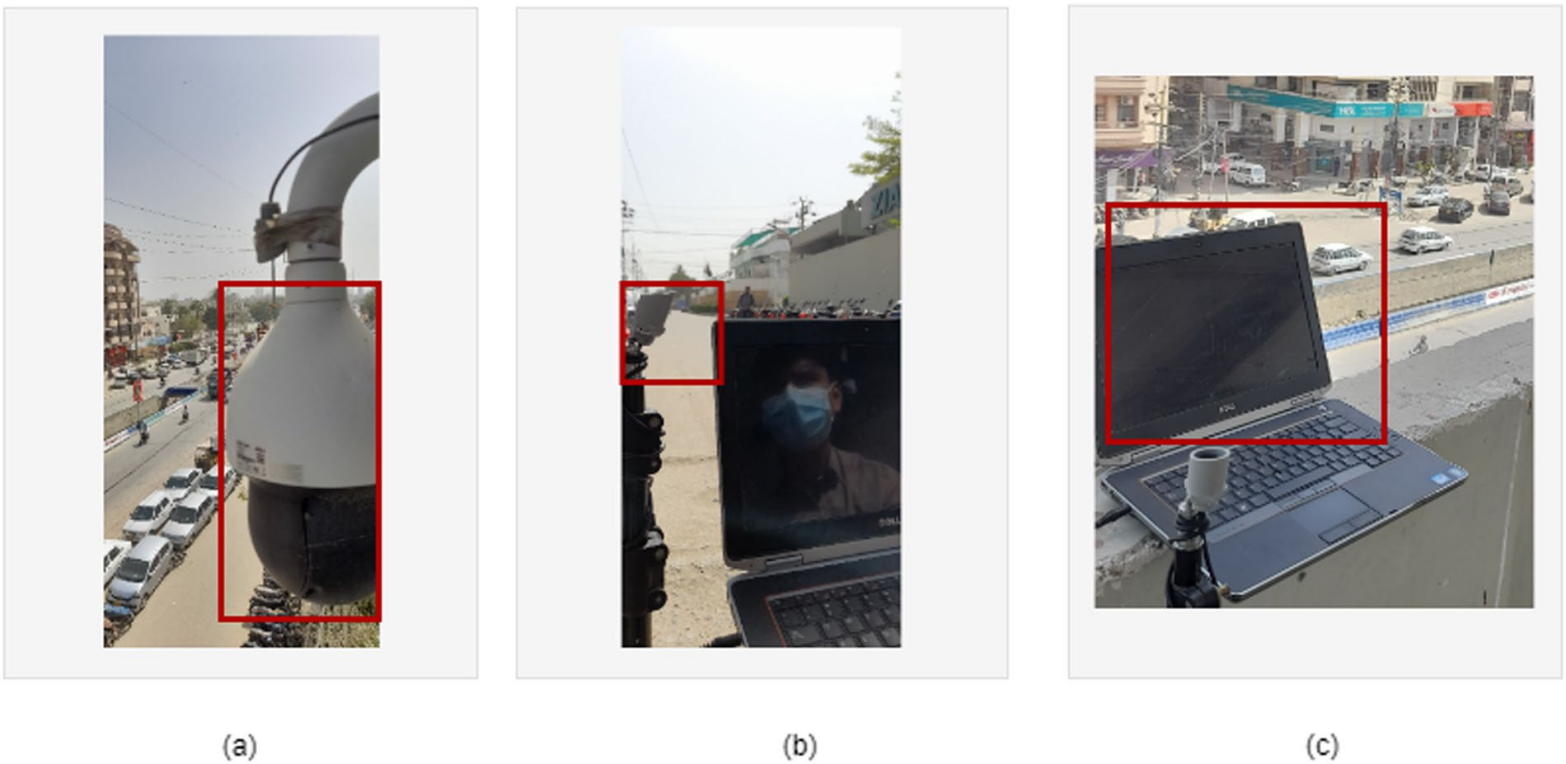
Various data recording devices used for database development: (**a**) showing IP-camera used for data collection; (**b**) showing mic used for data collection; (**c**) Laptop (i3 5th Generation) used for data collection. This figure represents various techniques and hardware used for data collection at different locations.

The camera used for data collection is independent of video quality and integrated with the sensitive and omnidirectional microphone sensor. Dahua and Hik-Vision cameras DH-SD59430U-HNI and Hik vision DS-2CD2T46G2–41 attached with ML-1WS ETS microphones were used for data collection as shown in Fig. 3. The ML-1WS microphone is a mono channel microphone with sensitivity of −43 db and with sound pressure level (SPL) of 120 db. The details of data collected from different mic integrated camera nodes are shown in Table 1.

**Table 1:** Detail description of data collected through ML1-WS audio sensor integrated with cameras.

Sensor ID	Format	Sampling Frequency	Variable Bit Rate (VBR)	Frequency Range	Operating Temperature	Sensor Pattern	Height at which audio sensor is installed
ML1-WS_0011	M4a	44100 Hz	199 kbps	50-16k Hz	34–35 C	Omni-directional	30 Meters
ML1-WS_0012	M4a	44100 Hz	199 kbps	50-16k Hz	34–35 C	Omni-directional	45 Meters
ML1-WS_0013	M4a	44100 Hz	199 kbps	50-16k Hz	34–35 C	Omni-directional	10 Meters
ML1-WS_0014	M4a	44100 Hz	199 kbps	50-16k Hz	32–33 C	Omni-directional	15 Meters

While collecting more specific data and in the real-world environment, ISO9001: 2015 and ISO 14001: 2015 certified emergency vehicle sirens were used for data recording. The audio sensor ML-1WS attached to the recording device (Dell i5, 3^rd^ Generation Laptop) shown in Fig. 3 has been used to record the emergency vehicle siren sound at multiple locations to different distance ranges. Total of five (5) mics were used, combined with IP-cameras and one with recording device. The recording device was placed at various distance points (10 to 30 meters from a stationary siren) to record ambulance vehicle siren sounds at various intensities. Similarly, the other road sound data were also recorded using the same hardware on the multiple streets of the city to get a wide range of road noises.

Since the process was cumbersome and required more effort and time for data acquisition, at last, the dataset was extended by searching for emergency sirens, and road noise sounds in videos and audios over the internet. Some of the data (audio files) were taken from the internet^7,19^ to enlarge the dataset as deep learning require larger dataset to get better results and accuracies^20,21^.

The complete breakdown of the database developed and collected through mixed methods is given in Table 2.

**Table 2:** Breakdown of the data collected through different methods.

Data Collection Methods	Emergency Vehicle Siren (Files)	Road Noises Without Emergency Vehicle Siren (Files)
Online datasets	100	110
Recording (Voice-Enabled Camera Nodes)	50	100
Recording (Experimental Setup)	750	690
Total	**900**	**900**

### Data filtration and cleaning

The collected data went through different filtration and segmentation processes. The sound recorded for the dataset development was in “m4a” and “mkv” formats. The sound files available on the internet were in mp3 and WAV format of different lengths and sampled at different frequencies. Therefore, the developed dataset must be uniform in format, sampling frequency, and length.

The length of all the files was clipped and set at 3 to 15 seconds. The different audio formats were converted into a single format: WAV, the standard format for further classification. At last, all the audio files were manually checked and played to remove the distorted audio samples.

According to Nyquist’s theorem, any periodic signal must be sampled at more than twice the maximum frequency found in an analogue signal. On analysing the data, it has been observed that the maximum frequency in the collected data set is 15 to 20 kHz. Thus, the sampling frequency should be at least twice that limit for better audio quality. 48 kHz is another standard audio sample rate^22–24^. The higher sample rate technically leads to more measurements per second and a closer recreation of the original audio, so 48 kHz is often used in “professional audio” contexts more than in music contexts. The definition of hi-res audio states that any music file recorded with a sample rate higher than 44.1 kHz is considered high definition (HD) audio^24^.Therefore, the sampling frequency of 48 kHz is selected. All data (1800 files) have a variable length ranging from 3 to 15 seconds, and the sound clips were of different sampling frequencies. Most of the data collected were substandard and of poor quality (sampling frequency below 48 kHz). Therefore, all audio files were up-sampled, and the data quality of the dataset was improved. It helps the system to achieve better results. In this way, developed data quality has increased to achieve better results.

The collected data was then converted into WAV format for feature extraction using Python’s Librosa library. Librosa library is used for audio analysis, providing the building blocks necessary to create an audio information retrieval system. All the “WAV” files have been sorted according to classes for feature extractions in the repository. Furthermore, twenty-six features have been extracted, including 21 Mel Frequency Cepstral Coefficients (MFCCs), the roll-off rate, zero-crossing rate, spectral centroid, spectral bandwidth, Chroma_stft using the Librosa library are also uploaded in a mentioned repository in CSV format, which is entirely ready to train classifier models. These features are used when working with audio data generation and Automatic Speech Recognition. It provides the building blocks necessary to create any audio information retrieval system.

### Final dataset

**Fig. 4:**
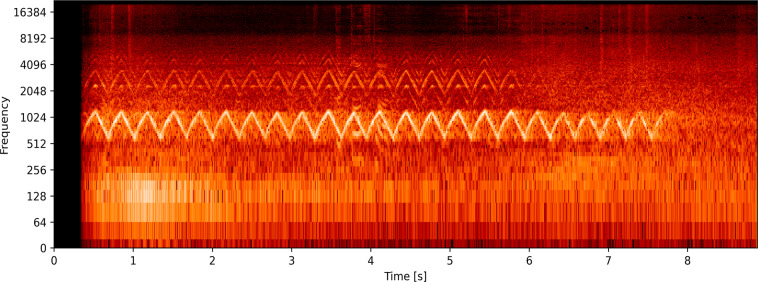
Spectrogram of emergency vehicle sirens showing frequency over time; This figure shows the spectrogram of emergency vehicle sirens in the time and frequency domain showing signal frequency over time.

**Fig. 5:**
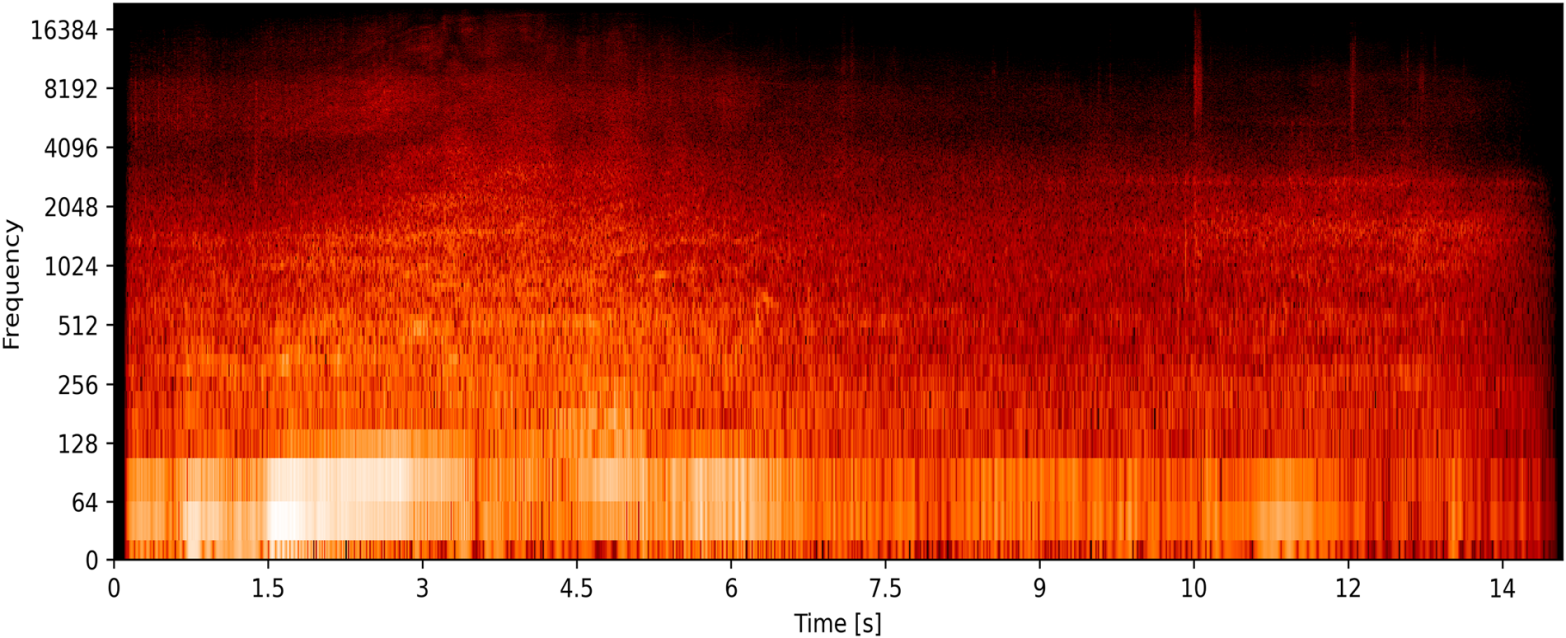
Spectrogram of different road noises found on roads in frequency domain; This graph shows the spectrogram of noises found on roads in frequency domain over time. It can be of different vehicle horns and real-world environment noises.

The dataset contains a total of 1800 audio files. These files are labelled into two classes, namely siren sound and road noise, and each of the classes contains 900 files of 3 to 15 seconds in length, as shown in Table 3. All the street noises are labelled in the same class, while the siren sound class has different types of siren voices like a wail, yelp, and two-tone siren. Another nine hundred audio clips merged in one class named Road Noises. Different spectrograms of the ambulance siren and other road noises are plotted using the python platform, as illustrated in Figs. 4 and 5, respectively. These spectrograms show the frequency components of emergency vehicle sirens and different road noises, representing the strength of the signal across time at different frequencies contained in a specific waveform. From these spectrograms, one can also visualized the highest frequency component found on roads.

**Table 3:** Overview of the data collected.

Dataset	Developed Dataset
Emergency Siren Sounds	900
Road Noise	900
Total No. of Sample	1800
Total Duration	3.15 Hours
Audio Clip Length	3–15 secs
Sampling rate	48 kHz

The primary objective of this data descriptor is to provide the scientific community a high-quality audio dataset of ambulance siren and road noises. The hustle free access to the dataset, which is publicly available, explaining the viewers; techniques from data collection to database development. However, for the ease of future researchers and to help other authors, all the required features which can be used for classification purpose, including 21 Mel Frequency Cepstral Coefficients (MFCCs), the roll-off rate, zero-crossing rate, spectral centroid, spectral bandwidth, and Chroma_stft, are extracted. They are gathered in the CSV file (Standard Format). These features are best known and widely used for audio recognition models. Especially MFCCs are the most perceptual scaling feature extraction from the audio signal, which is generally used as input to the AI-based models to produce better results and accuracies than other approaches.

It is the first dataset until acoustic monitoring of emergency vehicle sounds and traffic noises is fully available to the scientific community. This dataset can be used to develop different signal processing and deep learning methods for emergency siren detection on the city’s busiest streets to communicate with the rescue team for quick intervention. The dataset builds the path for additional research on the emergency siren, acoustic monitoring, and acoustic recognition of road vehicles. Researchers are more encouraged to implement different machine learning and deep learning techniques in their work due to accessibility and the larger dimension of this dataset.

## Data Records

The dataset recorded during this research work is uploaded at URL: 10.6084/m9.figshare.19291472 and the data are provided in two formats audio and text. The folders are named as “Emergency Vehicle Sirens” and the “Road Noises”, both contain the audio files and the CSV files are also named as same above. The audio files^25^ are in WAV format with a data size of around 2.7 GB, and the text data in CSV format, including the extracted features through which the Emergency siren and road noise could be classified using deep learning. Table 3 shows the summary of the complete dataset.

## Technical Validation

### Audio

The collected dataset from different locations recorded from the microphone used ML-1 WS from ETS is IP54 rated, part of a standard sound surveillance system. It has been used with specified electronics and distance limits as specified in the technical specification details from the manufacturer^26^. The ambulance siren used is S 300 W Wireless remote control ambulance siren. The siren used is ISO 9001:2015, ISO 14001:2015 CE, ECE and IP68 certificated to ensure quality. The siren has been used as per specification by the manufacturer^27^. Thus, making all the data validated according to the standard international parameters.

### Pre-processing

Each phase of audio dataset preparation was followed by a manual qualitative review of the audio data to assure data validity by listening to each sound. Furthermore, after the data harmonization process, a quantitative evaluation was undertaken to guarantee that the entire dataset had the exact parametric specification (i.e., the same sample frequency and formats have been used).

The trained and evaluated audio classifiers were pre-labelled subsets of the gathered audio data, beginning with raw WAV files and CSV files that had gone through the processing processes mentioned under the Data Processing section.

The raw audio recordings were manually categorized as ambulance sounds and road noises. Suppose there were audible road noises (such as conversation, movement, or a vehicle passing by sounds). Data from all hubs in a dataset was aggregated to produce more effective, diversified training and testing sets. The final distribution of noisy vs. ambulance files in each set was about equally divided, and a testing set was selected randomly from shuffled data using an 80/20 train/test split.

### Model evaluation

Table 4 shows the performance of the trained dataset on the Multilayer Perceptron algorithm along with the audio wav files and CSV format as a reference for the audio files utilized in the algorithm. Tables 5 and 6 represent the CSV format representation. Various features are extracted, such as frequency, the amplitude for the time-domain, and loading of the audio data, which decodes it into a 1-dimensional array which is a time series ‘y’ and sr is the time sampling rate of the ‘y’, by default sr is set to 22 kHz, then it is overridden by 48 kHz to enhance the data quality further. The data is divided into two formats for iterating and extracting its features: metadata and audio “Wav” files of the sounds using the Mel-Frequency Cepstral Coefficients.

**Table 4:** Overview of the data collected.

Sample Size		Accuracy
Train	Test	
300	76	0.90
500	125	0.945
1800	450	0.97

**Table 5:** Total of 26 Features of ambulance sirens.

File Name	Chroma_stft	Spectral Centroid	Spectral Bandwidth	Roll off rate	Zero Crossing rate	MFCC 1	MFCC 2
**ambulance142.wav**	0.345168054	0.30885303	1287.145377	1261.699532	2379.211895	0.081082858	74.00363159
**ambulance449.wav**	0.386695832	0.263406873	2223.479605	2115.848084	4718.463135	0.122690054	10.77275467
**ambulance888.wav**	0.517707944	0.281876475	1393.990435	1586.770873	2898.286321	0.064443735	52.15775681
**ambulance474.wav**	0.229988202	0.184972212	2211.761868	1923.778124	3913.245568	0.116267278	130.6637573
**ambulance305.wav**	0.149669617	0.115017645	1266.959915	1654.095586	1831.978666	0.094191331	213.1257172

**Table 6:** Total of 26 Features of different road noises found on roads.

File Name	Chroma_stft	Spectral Centroid	Spectral Bandwidth	Roll off rate	Zero Crossing rate	MFCC 1	MFCC 2
**road324.wav**	0.442203373	0.022000659	2545.346793	2426.47951	4754.28279	0.155189	−270.385
**road508.wav**	0.337758154	0.206504658	3470.430019	2706.420324	6899.114051	0.181979	11.29746
**road351.wav**	0.440022141	0.173754677	1248.230706	1904.995271	2556.032621	0.020921	−134.684
**road703.wav**	0.600493729	0.040601458	1075.899022	1827.481394	1814.793513	0.032874	−252.906
**road681.wav**	0.631075323	0.06535577	742.5482597	1372.708521	1231.119479	0.023869	−257.947

Table 5 shows all the extracted features of each audio sound (Ambulance Siren Class). The above table only shows two of the MFCCs, while 21 MFCCs are extracted during the feature extraction process. All these features are used to train the system and are unique for every audio file, helping the system to differentiate between the sounds. For instance, chroma stft represents the intensity of 12 distinctive pitch classes of any audio used for the study. At the same time, the spectral centroid is a quantity used to define a spectrum in digital signal processing. It shows where the spectrum’s centre of mass is located. It has a solid perception linked with the perception of a sound’s clarity. Similarly, Table 6 shows all the features of Road Noises compiled in CSV format. These features have identified labels, this data is in the form of categorical data, so this has to be changed by encoding the labels using Label Encoder.

The Encoded labels are target labels with values between 0 and 1. This transformer is used to encode target values. After pre-processing the data, this data is split into train and test datasets by the 80% & 20% ratios, respectively. The Multilayer Perceptron model is considered for the classification purpose^28,29^. A sequential model is used with input, hidden, and output layers as the respective layers have been applied to the model, such as the dense, dropout, and activation layers. The model builds upon thousands of training samples consisting of siren and non-siren audio. MLP is essentially a logistic regressor, implemented using a feedforward ANN, which uses features, extracted audio features in our case, to determine whether the input audio contains a siren or not, based on learned information.

**Fig. 6:**
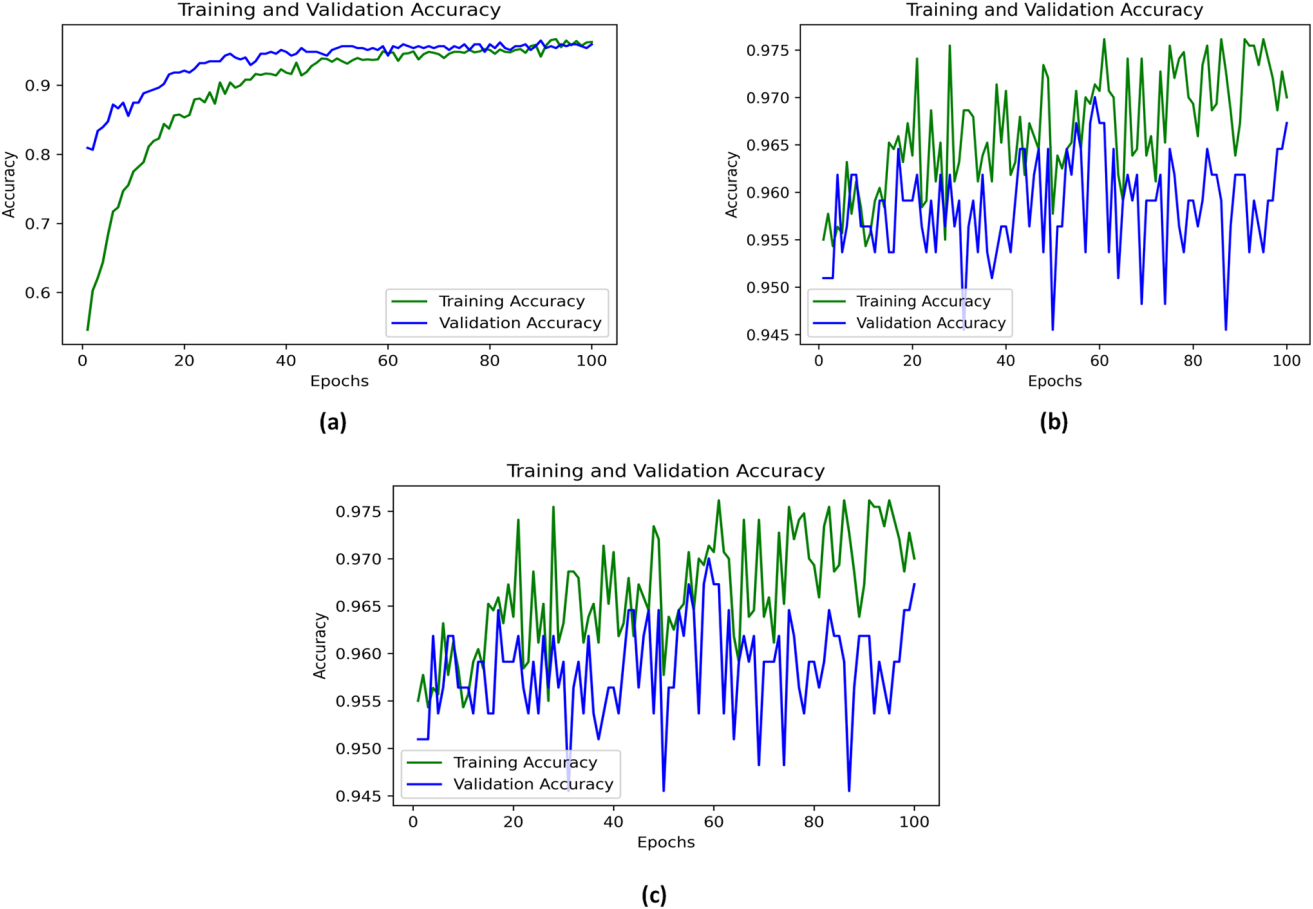
Evaluated the Trained and Validated Data percentage accuracies in this graph whereas (**a**) It shows the 90% accuracy, which is validated with the validation dataset; (**b**) It shows the 94% accuracy; (**c**) It shows the 97% accuracy.

The model evaluation plays a vital role in any artificial intelligence algorithm to measure the efficiency of a model during the training and validation phases. Figure 6, shows the model evaluation process has been done by using different evaluation metrics to understand the deep learning model performance by validating it. Figure 6(a) shows the evaluation of the 300 files of audio in total (Trained and Validated) by splitting ratios of 80% & 20%, which achieved 90% accuracy during the process, however in Fig. 6(b) which is trained & validated with the 1000 audio files in the total dataset achieved the 94% accuracy, and Fig. 6(c) achieved the 97% accuracy on the 1800 audio files for the model evaluation. This whole process shows that the increase in the dataset, the higher the accuracy and better performance of the model would be achieved.

## Data Availability

Code and the script files used to convert the sounds files into meaningful format are published in (https://github.com/tabarkarajab/Large-Scale-Audio-dataset-). We developed this code using Python and Pycharm Community software (Version 2021.3). The large-Scale Audio Dataset relies on the following dependencies: os, logging, traceback, shlex, and subprocess.
